# Bearing the Weight of End-of-Life Work: A Qualitative Study of Emotional Experience and Coping Among Palliative Care Professionals in Northern Italy

**DOI:** 10.3390/healthcare14152415

**Published:** 2026-08-05

**Authors:** Erika Iacona, Ines Testoni, Ciro De Vincenzo, Matteo Rigo, Stefania Zandonà, Daniela Delrio, Ivan Gallio, Alessandra Feltrin

**Affiliations:** 1 Department of Philosophy, Sociology, Education and Applied Psychology (FISPPA), University of Padua, 35131 Padua, Italy; erika.iacona@phd.unipd.it (E.I.); ines.testoni@unipd.it (I.T.); matteo.rigo@unipd.it (M.R.);; 2 Pain Therapy and Palliative Care Unit, Veneto Institute of Oncology IOV-IRCCS, 35128 Padua, Italy; 3 Hospital Psychology, Veneto Institute of Oncology IOV-IRCCS, 35128 Padua, Italy; alessandra.feltrin@iov.veneto.it

**Keywords:** palliative care, healthcare professionals, coping, compassion fatigue, compassion satisfaction, vicarious grief, meaning-focused coping, reflexive thematic analysis, supporting roles, end-of-life care

## Abstract

**Background**: Palliative care is a clinically, relationally and emotionally demanding field. Continuous exposure to suffering and death exposes professionals to compassion fatigue, vicarious trauma and grief, and burnout, while also offering opportunities for compassion satisfaction and meaning-making. How professionals cope is central to sustaining the supporting role in diseases with a poor prognosis. **Objectives**: To explore the emotional and experiential dimensions of palliative care work and the coping strategies, individual and team-based, that professionals deploy when accompanying patients and families at the end of life. **Methods**: Reflexive thematic analysis of 16 semi-structured interviews with palliative care professionals (5 physicians, 7 nurses, 3 psychologists, and 1 social-health worker; 14 women and 2 men) recruited from hospices, hospital wards and home-based services in Northern Italy. Sample size was guided by an information power model. Reporting follows the COREQ checklist. **Results**: Four themes were constructed: (1) the unsustainability of the workload; (2) the pleasure of care between empathic relationship, listening and welcoming; (3) the holistic role of the professional; and (4) the awareness of death. Across themes, participants mobilised a layered repertoire of problem-, emotion- and meaning-focused coping. Narratives fitting a structured moral-distress pattern were comparatively limited in participants’ accounts. **Conclusions**: Sustaining supporting roles in poor-prognosis care may require more than individual resilience; it depends on organisational support—structured supervision, reflective spaces, and communication and emotional training—that makes professionals’ largely informal coping work a shared and sustainable resource.

## 1. Introduction

Palliative care constitutes a clinically, relationally and ethically complex field of practice, characterised by the structured interaction of multiple professionals and the integration of medical, psychological, social and spiritual competences. Healthcare organisations are called upon to guarantee the patient’s right to care through a system capable of responding to multidimensional needs at the advanced and terminal phase of illness. In Italy, this is operationalised through Law 38/2010, which guarantees access to palliative care and pain therapy, and Law 219/2017 (Art. 5), which institutes Shared Care Planning—not merely a legal instrument but the outcome of a relational and decisional process founded on cooperation between professionals, patients and families.

Interdisciplinarity and multidisciplinarity are therefore not solely organisational arrangements but epistemological principles that orient palliative philosophy [1,2,3,4,5]. Shared communication, reciprocal listening between specialists and multidimensional needs assessment translate the principle of holistic care into an individualised plan aimed at quality of life and dignified accompaniment [6].

Yet the organisational and relational complexity of the palliative model does not exhaust itself in its structural dimension; it is profoundly mirrored in the subjective experience of the professionals involved. Working where suffering, vulnerability and proximity to death are everyday clinical realities entails intense and continuous emotional exposure [7] and locates professionals in a position of constant tension between professional responsibility, affective involvement and the structural limits of the care system. The literature has identified a family of constructs to describe the psychological impact of palliative work, including moral distress, vicarious trauma, vicarious grief, compassion fatigue and burnout, alongside compassion satisfaction [8,9,10].

### 1.1. The Psychological Toll of End-of-Life Work

Moral distress is defined as the psychological suffering arising when professionals recognise the morally appropriate course of action but cannot enact it because of institutional or relational constraints [11]. In palliative care, frequent exposure to bioethical dilemmas and conflicts between care objectives and organisational constraints amplifies this tension, contributing to powerlessness, frustration, reduced job satisfaction and emotional exhaustion [12,13].

Vicarious trauma refers to the psychological impact of repeated exposure to patients’ suffering and traumatic experiences. Healthcare professionals may undergo emotional and cognitive transformations linked to ongoing contact with pain, vulnerability and death [14,15], with possible alterations to schemas of meaning and a diminished sense of safety. Vicarious grief, while related, describes the bereavement experience indirectly lived by the professional following the repeated loss of patients, with symptoms analogous to those of direct loss [16]. Rando [17] distinguishes a predominantly empathic form, in which the professional shares the other’s pain without perceiving it as a personal loss, from a more identificatory form, in which the experience evokes prior personal losses and may destabilise the integrity of the self. Recent qualitative work on formal caregivers’ grief in end-of-life care underscores how such losses may accumulate across a professional’s career [18].

Sustained empathic engagement may also produce compassion fatigue—a state of psycho-emotional exhaustion arising from reiterated exposure to others’ suffering and manifested through chronic tiredness, irritability, cynicism, and a diminished sense of personal accomplishment [19,20,21]. Burnout, conceptualised as an occupational syndrome resulting from chronic work-related stress [22], is one of the most extensively studied consequences of occupational stress in palliative care nurses and physicians [10,23,24]. Conversely, compassion satisfaction—the perceived effectiveness and meaningfulness of helping work—is associated with emotional resilience, work–life balance and coherence between personal values and clinical practice [25]. Although these constructs overlap, they are analytically distinct: moral distress originates in constrained ethical agency; vicarious trauma and vicarious grief in the empathic internalisation of others’ suffering and loss; compassion fatigue in the gradual erosion of empathic capacity; and burnout in chronic organisational stress. Compassion satisfaction, by contrast, denotes the protective, generative counterpart of the same exposure. In what follows these are treated not as discrete diagnostic categories but as overlapping registers of a single emotional economy that professionals continuously regulate through coping.

### 1.2. Coping in the Supporting Role: A Conceptual Framework

How professionals respond to the dual exposure to vulnerability and meaning has been a central concern of the stress and coping tradition since Lazarus and Folkman [26], who defined coping as the constantly changing cognitive and behavioural efforts to manage specific external and internal demands appraised as taxing or exceeding personal resources. Their original distinction between problem-focused coping (acting upon the stressor or its conditions) and emotion-focused coping (regulating the emotional response to the stressor) has been progressively refined to include a third, meaning-focused dimension [27,28,29], which draws on values, beliefs and existential frames to reappraise stressful events and integrate them into a coherent narrative of self.

Meaning-focused coping is particularly relevant in end-of-life work. Park’s [29] meaning-making model—distinguishing global meaning (overarching beliefs, goals and sense of purpose) from situational meaning (the appraisal of a specific event)—captures the work of integration that practitioners undertake when accompanying patients whose trajectory cannot be cured. The same logic underpins meaning-centred approaches in palliative care [30,31] and self-care models for clinicians at the front line of end-of-life care [9,32].

Recent qualitative and mixed-methods studies suggest that palliative professionals deploy a layered repertoire of strategies: problem-focused work (boundary-setting, peer consultation, and structured handovers); emotion-focused regulation (temporary distancing, ritualised closure, and somatic self-soothing); and meaning-focused practices (reframing the caring relationship, valuing the present, and anchoring practice in personal and shared values) [9,33,34,35]. Whether these coping responses act as protective resources or as silent compensations for missing organisational support is an open question—and one with direct relevance for supporting roles in diseases with a poor prognosis.

### 1.3. Aims of the Present Study

Against this background, the present qualitative study explores how palliative care professionals in Northern Italy experience and ascribe meaning to their work, with a particular focus on the coping strategies—individual and team-based—they mobilise in the face of suffering, loss and death. The study seeks to: (i) describe the emotional and experiential dimensions of palliative work as lived by professionals across roles and settings; (ii) identify the problem-, emotion- and meaning-focused coping responses embedded in their accounts; and (iii) reflect on the organisational and educational conditions that sustain or undermine such responses, with implications for the supporting role in diseases with a poor prognosis. In doing so, the study makes three contributions. Empirically, it documents how professionals in different roles within the same multiprofessional teams assemble problem-, emotion- and meaning-focused strategies into an integrated and largely self-organised repertoire. Conceptually, it foregrounds meaning-focused coping—comparatively neglected in the palliative-workforce literature relative to burnout and compassion fatigue—as the register through which exposure to death is rendered generative rather than only depleting. Analytically, it treats the comparative muting of structured moral distress as a finding to be explained rather than assumed.

## 2. Materials and Methods

Reporting follows the Consolidated Criteria for Reporting Qualitative Research (COREQ) [36]; the full 32-item checklist is provided as Supplementary File S1.

### 2.1. Study Design and Theoretical Orientation

An interpretivist–constructivist epistemology was adopted, considered the most appropriate for exploring complex phenomena in their natural contexts and for understanding the meanings that participants attribute to their own experience [37]. Data were analysed by means of reflexive thematic analysis [38,39,40], an approach that conceptualises themes as analytic outcomes actively constructed by the researcher in dialogue with the data, rather than as patterns passively “emerging”.

### 2.2. Sampling and Recruitment

Purposive sampling with subsequent snowball expansion was used. An initial pool of professionals was identified through the research team’s institutional networks in hospices, hospital palliative care units and home-based services in Northern Italy. Inclusion criteria were: (i) being a healthcare professional currently working in a palliative care setting; (ii) at least six months’ continuous experience in the role; and (iii) capacity to consent and willingness to share lived experience in a research interview. No exclusion was applied on the basis of seniority or professional role, in order to capture the heterogeneity of perspectives operating within multiprofessional teams. Prospective participants were contacted individually by email or telephone by a member of the research team, given a written information sheet, and invited to take part; those who accepted were then asked to suggest further eligible colleagues, who were approached in the same way (snowball expansion). Recruitment thus combined purposive selection across roles and settings with participant-initiated referral, with all invitations issued directly by the research team.

Sample size was determined a priori in accordance with Malterud, Siersma and Guassora’s [41] information power model. Given (i) a narrow study aim focused on a specific professional experience, (ii) the use of theoretically informed, in-depth semi-structured interviews with high-quality dialogue, (iii) a heterogeneous yet bounded sample of informants with directly relevant expertise, and (iv) an analytic strategy based on in-depth case analysis rather than cross-case enumeration, a sample of approximately 15 participants was judged to carry sufficient information power. Recruitment continued until conceptual richness—assessed in iterative analytic meetings—was considered to have been achieved at 16 interviews. Consistent with reflexive thematic analysis, “saturation” was not treated as a fixed stopping rule; recruitment stopped when additional interviews were deepening and consolidating the existing organising concepts rather than generating new ones.

The final sample consisted of 16 professionals (14 women and 2 men; aged 27–58 years): 5 physicians, 7 nurses, 3 psychologists and 1 social-health worker. The inclusion of a single social-health worker reflects the structure of palliative care teams in the participating services, where this professional role is numerically smaller than nursing and medical roles; rather than aiming for cross-role generalisation, this participant was retained as an information-rich case offering a perspective complementary to those of clinical roles. Three participants reported less than one year of palliative care experience; the remaining thirteen had at least three years of service (overall mean = 7.8 years). Most participants reported previous experience in other healthcare departments, including emergency, onco-haematology, oncology and neurology. Two professionals approached during recruitment declined participation, citing time constraints; no participant withdrew after consent. Sample characteristics are summarised in Table 1.

### 2.3. Data Collection

Data were collected through individual semi-structured interviews between January and June 2023. The interview schedule (Supplementary File S2) was developed by the research team on the basis of the principal theoretical references concerning palliative work and covered four broad areas: (i) ethical and decisional experiences; (ii) the psychophysical impact of continuous exposure to suffering and death; (iii) the relational and emotional dimensions of care; and (iv) personal transformations, coping strategies and team-level resources. Open-ended questions were complemented by prompts emerging from participants’ responses, in order to favour expressive freedom and the deepening of significant aspects. The interview areas were derived deductively from the study’s guiding frameworks—the stress-and-coping tradition [26,27] and the palliative-workforce literature on moral distress, compassion fatigue and compassion satisfaction reviewed in Section 1—together with the team’s prior qualitative work on end-of-life care and loss [8,42], and were reviewed internally within the research team before use; no separate external pilot interviews were conducted.

Interviews were conducted online via secure video conferencing software by a member of the research team trained in qualitative interviewing and in psychosocial approaches to end-of-life care. No prior professional or personal relationship existed between the interviewer and the participants. Participants were informed of the interviewer’s institutional affiliation (University of Padua) and research focus on end-of-life care and were aware that the conversation was part of a study on professional experience in palliative care. Interviews lasted, on average, approximately one hour (range ≈ 45–90 min) and were audio-recorded with written informed consent. Brief reflexive field notes were taken immediately after each interview to record contextual impressions, salient affective tones and preliminary analytic ideas.

Interviews were conducted and transcribed in Italian, and the analysis was performed on the original Italian transcripts; the quotations presented in this article were translated into English by the authors, with translations reviewed within the research team to preserve the meaning and tone of the original.

Audio recordings were transcribed verbatim and anonymised in compliance with EU General Data Protection Regulation 2016/679; identifying details (institution names, patient references, and distinctive biographical features) were removed or paraphrased. Each participant was assigned a fixed pseudonym; in this manuscript, quotations are attributed by pseudonym and professional role, with pseudonyms and any contextual details chosen so as to minimise the risk of re-identification in a small sample drawn from a bounded geographical area.

### 2.4. Data Analysis

Analysis followed the six recursive phases of reflexive thematic analysis [38,39,40]: (1) familiarisation with the data through repeated reading of the transcripts and re-listening to the audio files; (2) generation of initial codes across the full dataset, line by line, with both semantic and latent codes; (3) construction of candidate themes by clustering codes around shared meaning; (4) review and refinement of themes against coded extracts and the full dataset; (5) definition and naming of themes, with each theme operationalised by a central organising concept; and (6) production of the analytic report. Reflexive thematic analysis was preferred over interpretative phenomenological analysis [43] because the aim was to construct cross-participant patterns of latent, theoretically informed meaning, linking emotional labour, meaning-making and coping across professional roles, rather than idiographic phenomenological accounts.

Initial coding was conducted by one member of the research team. Analytic meetings involving the research team—including senior researchers in palliative psychology, clinical psychology and Death Education—were held throughout the analysis to challenge, refine and reconfigure the developing thematic structure. The role of these meetings was not to verify “inter-coder reliability” in a positivist sense, which is incompatible with the epistemological commitments of reflexive thematic analysis, but to enrich interpretation and to surface taken-for-granted assumptions in the analytic process. Analysis was supported by manual coding of printed transcripts and structured analytic memos; no qualitative data analysis software was employed. Coding proceeded in a primarily inductive, data-driven manner; the coping framework (problem-, emotion- and meaning-focused coping) was applied as an interpretive lens during the later phases of theme development and definition, rather than as an a priori grid imposed on the raw data—an analysis best described as inductive at the coding stage and theoretically informed at the interpretive stage. The initial coding framework was granular and largely semantic (e.g., “bureaucratic overload”, “protective distancing”, and “waiting for the right moment”); the move to the four final themes was one of analytic abstraction and consolidation, in which early codes were merged, peripheral codes were set aside, and the coping function of each cluster was made explicit only at the theme-definition stage. This abductive movement between data and theory—inductive engagement combined with deductive attention to the sensitizing concepts of the coping framework—is well established in reflexive thematic analysis [44,45].

Although initial coding was conducted by one member of the research team, this is consistent with Braun and Clarke’s [39,40] explicit position that, in reflexive thematic analysis, multiple independent coders and coding-reliability metrics are neither necessary nor desirable, because coding is an interpretive act rather than a measurement to be verified. To guard against idiosyncratic readings, other team members independently read subsets of transcripts, proposed alternative interpretations, and specifically probed for potentially missing or silenced themes (e.g., overt conflict with colleagues or institutions); divergences were resolved by returning to the data rather than by computing agreement coefficients. Given its theoretical salience, material bearing on moral distress—situations in which participants recognised a right course of action but felt unable to enact it—was explicitly coded, so that the comparatively limited presence of such narratives reported in Section 4.2 rests on coding rather than on inference from silence. In these terms, the wider team functioned as “critical friends” [46], offering alternative readings that stress-tested the coherence and plausibility of the analysis rather than establishing inter-coder reliability.

Member checking was not pursued, consistent with Braun and Clarke’s [39,40] framing of reflexive thematic analysis; themes are analytic constructions rather than descriptive summaries of participants’ views, and their validity does not depend on participant endorsement. Quality and trustworthiness were instead pursued through prolonged engagement with the data, reflexive memoing, team-based analytic discussion, and the search for negative or divergent cases throughout coding. Trustworthiness was pursued through practices consistent with reflexive thematic analysis [47]: a reflexive journal, analytic memoing, and an audit trail documenting the evolution of codes and themes, alongside the team-based reflexive dialogue described above, so that rigour rested on reflexive engagement and transparency rather than on inter-coder reliability.

### 2.5. Reflexivity

The research team is composed of psychologists, psychotherapists and senior academics with extended scholarly and clinical engagement with end-of-life care, Death Education, and the psychosocial dimensions of suffering and loss. None of the team members held a clinical or supervisory role in the services from which participants were recruited at the time of the study. Team members hold predominantly humanistic and existential orientations towards care and have a background of empirical work documenting both the burden and the generative potential of working with dying patients. These positions informed analytic sensitivity to dimensions of meaning-making, dignity and relational ethics in participants’ accounts. Reflexive memos were used throughout to interrogate the influence of these positions on analytic choices, including in relation to potentially silenced themes (e.g., explicit conflict with colleagues or institutional anger).

### 2.6. Ethical Approval

The study was conducted in accordance with the Declaration of Helsinki and approved by the Ethics Committee for Psychological Research (Area 17)—Section of Psychology of the University of Padua (Protocol No. 5130, 9 January 2023). All participants provided written informed consent prior to the interview, including authorisation to audio-record the conversation. Confidentiality and the right to withdraw at any stage were re-stated at the start of each interview. Recruitment and data collection commenced only after this approval had been granted (data were collected between January and June 2023); no participant was approached or interviewed beforehand.

## 3. Results

Reflexive thematic analysis produced four themes: (1) the unsustainability of the workload; (2) the pleasure of care between empathic relationship, listening and welcoming; (3) the holistic role of the professional; and (4) the awareness of death. Each theme is illustrated below with verbatim extracts. The thematic structure, including sub-themes and the coping functions activated within each, is summarised in Table 2.

### 3.1. The Unsustainability of the Workload

Across roles, participants consistently framed their work in terms of workload overload and structural under-resourcing of the national health service. The increase of caring demands had not been matched by an increase in staffing or facilities.

“This is just the kind of historical moment we are in: there is a shortage of doctors, of nurses, of facilities, of money to purchase materials. The context itself is like this. But we have been like this for years, even in the good years.”

—*Sara, Nurse*

Senior professionals in particular highlighted the increase in administrative and digital tasks, framed as an additional and largely invisible burden encroaching on the time available for direct care.

“The workload has truly quadrupled, and then there is the technology-data collection, articles we are asked to provide, things to deliver in person, a whole series of computer-based activities, forms and so on, which, I repeat, costs us effort.”

—*Sara, Nurse*

“The bureaucratic part of the work has worsened, because when I started in this service there were no computers. […] You have to enter everything into the computer. The indirect time of care has tripled.”

—*Anna, Nurse*

Crucially, participants linked administrative load to the erosion of direct patient time. The salient emotional register was indignation—not at patients or colleagues but at a system perceived as misallocating professional resources.

“The one thing that really makes me angry is this: why must we be so squeezed, sometimes performing tasks that are not ours, such as secretarial work or answering the telephone? […] If I am here doing a consultation, just as I would want for myself, I would not want the doctor to be distracted by a phone call.”

—*Laura, Palliative care physician*

Coping in this theme was largely confined to problem-focused micro-strategies (pacing the work, prioritising direct care, and signalling overload to colleagues) and to emotion-focused responses such as indignation, which functioned as a protest against role distortion rather than as a means of changing it.

### 3.2. The Pleasure of Care: Empathic Relationship, Listening and Welcoming

Despite the overload, participants converged on the centrality of the relationship with patients and families as the core of their professional practice. Active listening and compassionate welcoming emerged as fundamental relational competences.

“The conversation is the basis of everything, both with the patient and with the family; you really must be empathic, dialogue with them, have a great deal of patience.”

—*Elena, Nurse*

“I try to inform compatibly with the receptive capacity of the patient or the family, so as to welcome them and to guide them towards the best choice for the well-being of the patient and the family.”

—*Marco, Palliative care physician*

In the home-care setting, the relationship took on further layers of complexity: the professional entered a private space often traversed by tension, anger and unprocessed suffering. The dominant coping response was a deliberate, silent containment of the family’s affect.

“We arrive at their home, so the ground is theirs, and we come and acknowledge that they have been mistreated, and they pour out on us their anger, disappointment, bitterness and pain, and you have to stand there in silence because that is the time for listening. Sometimes you feel uncomfortable because they are right, but as a professional you have to maintain a professional position.”

—*Sara, Nurse*

Regulating empathic involvement recurred across accounts as a learned competence rather than as a personality trait. Participants described a progressive titration of closeness and distance—what the coping literature would describe as a combination of emotion-focused (protective distancing) and meaning-focused (preserving the helping stance) work.

“I am empathic, because this work draws me in. Once I was much more so-I would almost merge with it, it was too much… you get drawn in, you grow sad, it weighs on you too much. I have learnt that I must not merge with the patient’s suffering, because then I suffer too; I can be useful when I am there with them, but I cannot carry it home.”

—*Anna, Nurse*

“There must always be a boundary, both for self-protection and for reasons of role […] yet, given the way I am, involvement is still important.”

—*Daniela, Physician*

“It was not as if I could cry in front of that poor woman—that was not what she needed; then outside I had a little cry, I texted my partner and let it out.”

—*Alessia, Psychologist*

#### Team Support as an Informal Coping Resource

Within the team, most participants described collaborative and supportive relationships. Peer consultation operated as the principal—and often the only—vehicle for processing complex situations.

“If I am in difficulty […] I ask a colleague for help. […] ‘come into the consulting room with me, let us see this patient together; tell me what impression you have, let us share this.’ Generally I look for cooperation.”

—*Laura, Palliative care physician*

“I think this is the essential way to make certain situations less heavy, also because we have nothing structured-no psychological support, no groups; and even team meetings are currently not structured to give voice to these lived experiences.”

—*Daniela, Physician*

Team relationships were not, however, uniformly protective. One participant described the team as the very setting in which the regulation of involvement, otherwise achieved with patients, failed:

“Personal involvement in contact with the patient, hardly ever-most of the time I manage to keep the right distance; with the team I work with, on the other hand, no.”

—*Beatrice, Psychologist*

The interpersonal dimension of the team thus functioned as an informal—though not uniformly protective—coping resource, partially compensating for the absence of formalised supervision spaces.

### 3.3. The Holistic Role of the Professional

A third theme concerned the centrality of the patient as a multidimensional person, rather than as a bearer of symptoms. Participants framed their work as continuously oriented towards the well-being of the ill person, the personalisation of the pathway, and the safeguarding of dignity in the terminal phase. This orientation operated as a stable meaning-focused anchor for practice.

“My attitude is exactly what I feel as morally required: that of being an instrument for him, in order to give this last moment of life the sense of value, also as a person.”

—*Laura, Palliative care physician*

“I act above all in trying to understand how I can best help the patient and personalise the pathway […] trying to mediate between what can be given and what the patient needs to receive […] I put all my energy and commitment into doing the best I can for the patient.”

—*Giulia, Nurse*

Respect for the patient’s times, will and wishes was repeatedly framed as an ethical discipline; rather than imposing a clinical tempo, professionals described attuning to the tempo dictated by the illness and the person.

“It is the illness that always dictates the situation […] you also have to wait for the right moment to say things or to do certain things […] there are ways and times that have to be respected.”

—*Anna, Nurse*

“We try to enter the homes respecting the situations we find, with the utmost delicacy.”

—*Maria, Nurse*

“I always keep clearly in mind that I have before me people who sometimes ask me for a dressing change but at other times need to be listened to […] you have to be there to seize the moment.”

—*Maria, Nurse*

Overall, the holistic role functioned as a moral anchor that prevented the workload pressures described in Section 3.1 from collapsing the meaning of the work into its tasks; even when overwhelmed, participants retained a clear narrative of why their work mattered. Reading through the coping framework, this holistic orientation functions as meaning-focused coping rather than as a description of good clinical practice alone; maintaining a clear account of why the work matters is itself a strategy that buffers the strain documented in Section 3.1.

Professional roles contributed complementary perspectives to holistic care, with participants emphasising the importance of role clarity within multidisciplinary teams.

“I think it is important that the psychologist’s role within the multidisciplinary team is clearly understood.”

—*Alessia, Psychologist*

### 3.4. Awareness of Death

Across roles, participants described a continuous, low-grade awareness of mortality as an inescapable feature of their work. Proximity to dying patients did not stay confined to the workplace; it reshaped their relationship with their own life, time and priorities.

“It has confirmed to me that death can come when you least expect it […] so you have to try to savour life as much as possible […] Living for many years in contact with death has changed me a little.”

—*Laura, Palliative care physician*

“Daily contact keeps the idea in a corner of your head that it could happen.”

—*Maria, Nurse*

For some participants, identification with the dying—particularly with patients whose biographical features (age, family composition, and illness trajectory) resonated with their own—rendered thoughts of death intrusive and difficult to contain.

“You do think about death, even though you hope it will come as late as possible, because you put yourself in the other person’s shoes; it makes you think it could happen to you, how stressful it would be […] then you try to set the thought aside, but it is not easy.”

—*Elena, Nurse*

Other participants described the maturation of a more integrated relationship with mortality—a form of personal growth in which exposure to dying yielded a more deliberate and present-focused way of living. In the coping literature, this corresponds to meaning-focused, integrative responses akin to post-traumatic and existential growth.

“Here, suffering is a daily reality, deaths are a daily reality, so yes, I have matured an awareness [of death] and it helps me to live better.”

—*Sara, Nurse*

The two trajectories—intrusive identification on the one hand, integrative growth on the other—co-existed across the sample and often within the same account, suggesting that awareness of death is not a stable state but a recurrent appraisal that can move between distress and meaning depending on the situational and team-level resources available at a given time.

## 4. Discussion

Take-home message. Palliative care work is sustainable not because individual professionals are exceptionally resilient but because they continuously assemble—largely on their own—a layered repertoire of coping responses that integrates problem-focused micro-strategies, emotion-focused regulation and meaning-focused reframing. Where organisational “containers” (structured supervision, reflective spaces, communication and emotional training) are absent, this coping work is carried by informal team relationships and by the personal moral commitment of professionals. The implication is direct: investing in formal containers does not replace the meaning-making core of palliative work but protects it from being silently subsidised by individual wear-and-tear.

Three features distinguish these findings from the existing palliative-workforce literature. First, whereas that literature has concentrated on burnout and compassion fatigue as outcomes to be prevented, our data foreground meaning-focused coping as the central process through which exposure to death becomes generative. Second, by sampling across roles within the same teams, the study shows that this coping repertoire is assembled collectively and informally, in the near-total absence of structured organisational support. Third, the comparative muting of structured moral distress—discussed below—is itself a counter-expected observation in a setting usually assumed to be saturated with ethical conflict.

### 4.1. Reading the Themes Through a Coping Lens

The first theme—the unsustainability of the workload—is consistent with the literature on compassion fatigue, which identifies prolonged overload and continuous exposure to suffering as risk factors for emotional exhaustion [21] and burnout [10,23,24]. Participants reported symptoms compatible with work-related stress, while maintaining clinical attentiveness. The coping repertoire mobilised in this theme was predominantly problem-focused (pacing and prioritising direct care), but its leverage was bounded by structural constraints that the individual could not modify; indignation operated as an emotion-focused protest rather than as a coping resolution. This resonates with a growing critique of the invisibility of relational and nursing work in conventional organisational metrics: cognitive, relational and coordination activities central to care delivery are systematically under-represented in administrative and information systems, and standardised nursing data and terminologies have been proposed as one means of rendering this complexity visible [48]. Palliative care, where much of the work is relational and anticipatory, is a paradigmatic setting for this mismatch between what is done and what is counted.

The second theme—the pleasure of care—illustrates the compensatory role of the caring relationship. Consistent with Figley’s [49] model, compassion satisfaction appears as a resource that offsets, without eliminating, the costs of emotional labour [50]. The positive feedback received from patients and families carries a markedly protective value, especially in the absence of adequate institutional recognition [34,35]. The regulation of empathic involvement described by participants is precisely the work of emotion-focused coping: a learned, dynamic titration of closeness and protective distance through which the helping stance can be sustained over time [9,33]. In these terms, the modulation of empathic involvement participants described is a form of emotional labour in Hochschild’s sense [51], governed here not by commercial display rules but by the ethical imperative to sustain a compassionate yet bearable presence.

The third theme—the holistic role of the professional—operates at a meaning-focused level. Participants’ adherence to person-centred care principles [52] and their emphasis on communication as a genuine “time of care” [53,54] reflect a global meaning framework [29] in which the work is morally intelligible even when its conditions are difficult. This anchor prevents workload pressure from collapsing the work into mere tasks and is a key reason why participants describe themselves as committed despite their fatigue.

The fourth theme—awareness of death—most clearly enacts meaning-focused coping. Vicariously experienced death does not produce only suffering; in line with Varga and Gallagher [55] and with meaning-centred approaches in palliative psychology [30,31], it can foster a re-evaluation of the meaning of existence and a more present-oriented stance towards life. The coexistence of intrusive identification and integrative growth within the same accounts mirrors the dialectic between vicarious trauma [14,15] and post-traumatic growth long described in the bereavement literature. The integrative-growth trajectory, in particular, is consistent with the post-traumatic-growth framework [56], in which sustained confrontation with mortality can catalyse a re-ordering of priorities and a deepened appreciation of life.

A note on professional roles. Although nurses, physicians and psychologists occupy distinct relational and emotional positions in end-of-life care—nurses through sustained bodily and domestic proximity, physicians through decisional and prognostic responsibility, and psychologists through an explicitly containing function—these differences remained in the background of a dataset organised around shared, cross-role experiences. Psychologists highlighted the importance of role clarity and integration within multidisciplinary teams (Section 3.3). The convergence we observed should not be read as evidence that role is immaterial; the small number of participants per role precludes systematic role comparison, which we flag as a priority for future, role-stratified research (Section 4.4).

### 4.2. A Distinctive Finding: Why Is Structured Moral Distress So Muted?

A notable feature of participants’ accounts is the comparatively limited presence of narratives reducible to structured moral distress [11,12,13]. Participants reported a strong and consistent commitment to suspending personal judgements and aligning clinical decisions with patients’ wishes; explicit conflict with institutions, colleagues or families was rare in the corpus. We suggest three, non-mutually exclusive, interpretations.

First, this may reflect the maturation of the Italian palliative care system after Law 38/2010 and Law 219/2017, which has consolidated patient self-determination and Shared Care Planning as institutional defaults. The space in which moral distress classically arises—the recognition of the “right action” followed by the inability to enact it—may be narrowed, in palliative care specifically, by a legal–clinical architecture that explicitly authorises the privileging of patient values. Second, the voluntary nature of recruitment may have over-represented professionals whose adaptation to the role has already integrated moral conflict into a stable framework of meaning; professionals struggling with unresolved moral distress may have been less inclined to participate. Third, the muting of moral distress may itself be a coping achievement, sustained by the team-level and meaning-focused work described above; in this reading, the apparent absence is a sign of effective, but fragile, integration rather than of objective absence. Understood in this way, the muting of structured moral distress is closer to moral resilience—the capacity to preserve integrity amid moral adversity [57]—than to moral disengagement or indifference.

Importantly, this does not mean that ethical tension is absent; communicative difficulty, decisional conflict and breaking bad news continue to mobilise moral tensions [8]. What is muted is the structured, paradigmatic moral distress narrative—and that itself is an analytic finding worth pursuing. We therefore refrain from claiming that moral distress is objectively less prevalent in this setting; our data can establish only that the structured moral-distress narrative was comparatively muted in what participants chose to report—an observation compatible with both genuine contextual attenuation and social-desirability or self-selection effects.

### 4.3. Implications for Supporting Roles in Diseases with a Poor Prognosis

Reading alongside the scope of this Special Issue, the findings translate into four concrete implications for the design and sustainability of supporting roles in poor-prognosis care.

Organisational level. The most consistent gap reported by participants was the absence of formalised supervision and psychological support. Hospice and palliative care services should institutionalise (a) regular reflective practice groups facilitated by a trained clinician external to the unit, (b) post-event debriefing protocols for clinically and emotionally critical cases, and (c) supervision time recognised as work time rather than as discretionary or after-hours activity. Without this, peer support—currently the main informal container—will continue to operate as a silent subsidy provided by senior staff to the system.

Educational level. Training curricula for palliative care professionals should explicitly include communication skills (especially around breaking bad news and prognostic uncertainty), emotional regulation, and meaning-focused approaches to self-care [9,32,53]. Where possible, these competences should be taught and assessed alongside clinical skills, signalling their professional rather than personal status.

Workload and role design. The encroachment of administrative and digital tasks on direct care time was a major and remediable source of strain. Auditing the administrative load carried by clinicians, delegating non-clinical tasks to dedicated administrative staff where feasible, and protecting direct-care time would have a measurable effect on the emotional sustainability of the role.

Cultural recognition. Several participants framed positive feedback from patients and families as a more powerful protective resource than formal recognition by the institution [34,35]. Aligning institutional recognition with the actual moral economy of the work—for instance, through structured acknowledgement of complex cases, patient/family stories, or career pathways that value longevity in palliative care—is a low-cost, high-symbolic-yield intervention.

### 4.4. Strengths and Limitations

Strengths of the study include the explicit theoretical framing through the coping literature, the COREQ-compliant reporting (Supplementary File S1), the diversity of professional roles included, and the attention to reflexivity and to the active construction of themes rather than to their alleged “emergence”. Several limitations qualify the findings. First, recruitment was voluntary and may have favoured professionals more inclined to reflexive engagement, potentially under-representing those experiencing more acute distress, disengagement or unresolved moral conflict; this is particularly relevant for the interpretation of the muted moral distress narrative (Section 4.2). Second, the sample was geographically bounded to Northern Italy and to services operating within a specific legal and organisational architecture; transferability to different healthcare systems and cultural contexts should not be assumed without comparative work. Third, the inclusion of a single social-health worker constrains the ability to develop role-specific claims for this professional figure and signals an area for purposive expansion in future research. Fourth, the cross-sectional design captures lived experience at a single point in time and cannot trace the evolution of coping strategies and emotional patterns across career trajectories. Fifth, no data triangulation with observational, organisational or quantitative measures was undertaken; combining interviews with brief validated measures of burnout, compassion fatigue and compassion satisfaction (e.g., the Professional Quality of Life Scale) would strengthen future mixed-methods designs. Sixth, social desirability—especially given the moral salience of the topic—cannot be excluded. Seventh, as a semi-structured interview study, the framing and ordering of questions will have shaped what participants foregrounded; a schedule emphasising coping and meaning may itself have elicited more coping-oriented and less conflict-oriented accounts. Finally, participants’ emotional burden may arise not only from exposure to death and dying but also from caring for patients with highly complex, multidimensional needs; patient-related complexity of nursing care is an established construct [58] that the present design did not measure, and future research should examine the relationship between care complexity and professionals’ emotional experience, distinguishing the burden of mortality exposure from that of clinical-relational complexity. Relatedly, although four professional roles were sampled, nurses and physicians contributed most of the interview material, so the illustrative quotations are weighted towards these roles while the psychologist and social-health-worker perspectives are comparatively under-represented; role-stratified sampling is therefore needed to substantiate role-specific claims.

## 5. Conclusions

Our findings suggest that working in palliative care is not well captured by the notion of simply “doing a difficult job well”; participants described it as a continuous, layered work of coping in which problem-focused micro-strategies, emotion-focused regulation and meaning-focused integration are assembled, often invisibly, by professionals and informal teams. The four themes constructed in this study—the unsustainability of the workload, the pleasure of care, the holistic role of the professional and the awareness of death—describe both the costs and the generative potential of this work.

Sustaining the supporting role in diseases with a poor prognosis may benefit from, in addition to individual resilience, an organisational infrastructure that converts this private coping work into a shared and replenishable resource. Structured supervision spaces, reflective practice groups, training in communication and emotional skills, an audit of administrative load and institutional recognition of the moral economy of palliative work are potential organisational measures. Future research should adopt longitudinal and multi-site comparative designs, expand the inclusion of underrepresented professional figures (social-health workers, chaplains, and volunteers), and triangulate qualitative accounts with validated measures of professional quality of life. The aim is not only to describe the burden of end-of-life work but to inform the systems that make accompanying patients and families at the end of life a sustainable professional and human practice.

## Figures and Tables

**Table 1:** Participant characteristics (N = 16).

Characteristic	Categories	*n* (%)
Gender	Women	14 (87.5)
	Men	2 (12.5)
Age (years)	Mean (range)	≈42 (27–58)
Professional role	Physicians	5 (31.3)
	Nurses	7 (43.8)
	Psychologists	3 (18.8)
	Social-health worker (OSS)	1 (6.3)
Care setting (current)	Hospice (inpatient)	-*
	Hospital palliative care unit	-*
	Home-based palliative care	-*
Experience in palliative care	<1 year	3 (18.8)
	≥3 years	13 (81.2)
	Mean (years)	7.8
Prior experience in other settings	Emergency, oncology, onco-haematology, neurology, etc.	Most participants

* Several participants worked across more than one setting; exact distributions are not reported to minimise the risk of re-identification in a small, regionally bounded sample.

**Table 2:** Themes, sub-themes, coping function and illustrative quotations.

Theme	Sub-Theme	Coping Function	Illustrative Quote
**1. Unsustainability of the workload**	Chronic staffing shortages and structural under-resourcing	Problem-focused (limited): pacing the work; signalling overload	“There is a shortage of doctors, of nurses, of facilities […] but we have been like this for years.” (Sara, Nurse)
	Bureaucratic and digital overload	Problem-focused: prioritising direct care over admin tasks	“The indirect time of care has tripled.” (Anna, Nurse)
	Encroachment on direct patient time	Emotion-focused: indignation as protest against role distortion	“The only thing that really makes me angry is this: why must we be so squeezed […]” (Laura, Palliative care physician)
**2. Pleasure of care: empathy, listening, welcoming**	The caring relationship as core of the work	Meaning-focused: anchoring practice in relational value	“The conversation is the basis of everything […] you really must be empathic, dialogue with them, have a great deal of patience.” (Elena, Nurse)
	Entering the family’s private space (home care)	Emotion-focused: silent containment of others’ anger and pain	“We arrive at their home, so the ground is theirs […] you have to stand there in silence because that is the time for listening.” (Sara, Nurse)
	Regulating empathic involvement	Emotion-focused: boundary-setting; protective distancing	“I was much more so […] I cannot carry it home.” (Anna, Nurse)
	Team support as an informal coping resource	Problem-focused: peer consultation, shared decision-making	“I think this is the essential way to make certain situations less heavy […] we have nothing structured, no psychological support.” (Daniela, Physician)
**3. Holistic role of the professional**	Patient at the centre, multidimensional needs	Meaning-focused: aligning practice with patient-centred values	“My attitude is exactly what I feel as morally required: that of being an instrument for him […]” (Laura, Palliative care physician)
	Personalisation and mediation of the pathway	Problem-focused: tailoring care; brokering between options and constraints	“I act above all in trying to understand how I can best help the patient and personalise the pathway.” (Giulia, Nurse)
	Respect for the patient’s times and wishes	Meaning-focused: attunement to the illness’ tempo as ethical practice	“It is the illness that always dictates the situation […] there are ways and times that have to be respected.” (Anna, Nurse)
**4. Awareness of death**	Reflective re-evaluation of life	Meaning-focused: integrating finitude into a coherent life narrative	“Death can come when you least expect it […] you have to try to savour life as much as possible.” (Laura, Physician)
	Identification with the dying as a source of distress	Emotion-focused: cognitive distancing; deliberate ‘setting aside’	“You put yourself in the other person’s shoes […] then you try to set the thought aside, but it is not easy.” (Elena, Nurse)
	Death as occasion for personal growth	Meaning-focused: post-traumatic/existential growth	“Here, suffering is a daily reality, deaths are a daily reality, so yes, I have matured an awareness [of death] and it helps me to live better.” (Sara, Nurse)

## Data Availability

The qualitative data generated and analysed during the present study are not publicly available due to confidentiality and ethical restrictions, but anonymised excerpts and the codebook can be obtained from the corresponding author upon reasonable request and subject to ethical review.

## Supplementary Materials

The following supporting information can be downloaded at: https://www.mdpi.com/article/10.3390/healthcare14152415/s1, Supplementary File S1—COREQ 32-item Checklist [36]; Supplementary File S2—Semi-structured Interview Guide.
