# Supplementary material for: Bearing the Weight of End-of-Life Work: A Qualitative Study of Emotional Experience and Coping Among Palliative Care Professionals in Northern Italy
Source: Healthcare (Basel). 2026 Aug 5;14(15):2415. doi: 10.3390/healthcare14152415 (PMC13464988; doi:10.3390/healthcare14152415)
Supplement: Supplementary file 1 [file healthcare-14-02415-s001.zip › Supplementary File S1_COREQ_Checklist.pdf]

Supplementary File S1 — COREQ Checklist

"Bearing the Weight of End-of-Life Work: A Qualitative Study of Emotional Experience and Coping Among Palliative Care Professionals in Northern Italy"

Consolidated Criteria for Reporting Qualitative Research (COREQ; Tong, Sainsbury & Craig, 2007). A 32-item checklist for interviews and focus groups. 'Reported on' refers to sections of the revised manuscript and to supplementary files.

Reference: Tong, A., Sainsbury, P., & Craig, J. (2007). Consolidated criteria for reporting qualitative research (COREQ): A 32-item checklist for interviews and focus groups. International Journal for Quality in Health Care, 19(6), 349–357. https://doi.org/10.1093/intqhc/mzm042

#	Item	Response	Reported on
**DOMAIN 1: Research team and reflexivity**			
Personal characteristics			
**1**	Interviewer/facilitator: Which author/s conducted the interview or focus group?	Interviews were conducted by a member of the research team (S.Z) trained in qualitative interviewing and in psychosocial approaches to end-of-life care.	*Methods §2.3 — Data collection*
**2**	Credentials: What were the researcher's credentials? (e.g., PhD, MD)	The research team comprised members with backgrounds in Psychology and related disciplines, with qualifications ranging from undergraduate and master's degrees to PhD level, and including a licensed psychologist-psychotherapist and a senior academic. The interviewer was a master's-level researcher in Psychology at the time of the study.	*Title page; Notes on contributors*
**3**	Occupation: What was their occupation at the time of the study?	All authors were affiliated with the FISPPA Department, University of Padua, or with the Veneto Institute of Oncology IOV-IRCCS, in research and clinical roles related to palliative psychology, clinical psychology, and Death Education.	*Title page*
**4**	Gender: Was the researcher male or female?	The research team comprised both women and men. The interviewer was female.	*Methods §2.5 — Reflexivity*
**5**	Experience and training: What experience or training did the researcher have?	The team has extended academic and clinical engagement with palliative psychology, Death Education and the psychosocial dimensions of suffering and loss, and has training in semi-structured qualitative interviewing and reflexive thematic analysis.	*Methods §2.5 — Reflexivity*
Relationship with participants			
**6**	Relationship established: Was a relationship established prior to study commencement?	No. No prior professional, clinical or supervisory relationships existed between the interviewer and the participants at the time of the interviews.	*Methods §2.3 — Data collection*
**7**	Participant knowledge of the interviewer: What did participants know about the researcher?	Participants were informed of the interviewer's institutional affiliation (University of Padua), research focus on end-of-life care, and disciplinary training (psychology).	*Methods §2.3 — Data collection*
**8**	Interviewer characteristics: What characteristics were reported about the interviewer/facilitator?	Disciplinary background (psychology), academic affiliation, training in qualitative interviewing, and absence of clinical role within the recruiting services were disclosed to participants.	*Methods §2.5 — Reflexivity*
**DOMAIN 2: Study design**			
Theoretical framework			

**9**	Methodological orientation and theory: What methodological orientation was stated to underpin the study?	Interpretivist–constructivist epistemology. Analytic approach: reflexive thematic analysis (Braun & Clarke, 2006, 2019, 2021).	*Methods §2.1 — Study design and theoretical orientation*
Participant selection			
**10**	Sampling: How were participants selected?	Purposive sampling with subsequent snowball expansion, through the research team's institutional networks in palliative care services in Northern Italy.	*Methods §2.2 — Sampling and recruitment*
**11**	Method of approach: How were participants approached?	Eligible professionals were contacted by email or telephone by a member of the research team. Subsequent participants were referred by colleagues who met the inclusion criteria.	*Methods §2.2 — Sampling and recruitment*
**12**	Sample size: How many participants were in the study?	Sixteen participants (14 women, 2 men; aged 27–58): 5 physicians, 7 nurses, 3 psychologists, 1 social-health worker.	*Methods §2.2 — Sampling and recruitment; Table 1*
**13**	Non-participation: How many people refused to participate or dropped out?	Two professionals approached during recruitment declined to participate, citing time constraints. No participant withdrew after providing consent.	*Methods §2.2 — Sampling and recruitment*
Setting			
**14**	Setting of data collection: Where was the data collected?	All interviews were conducted online via secure video conferencing software. Participants joined from a familiar private environment (typically home or workplace).	*Methods §2.3 — Data collection*
**15**	Presence of non-participants: Was anyone else present besides participants and researchers?	No. Interviews were conducted one-to-one in private.	*Methods §2.3 — Data collection*
**16**	Description of sample: What are the important characteristics of the sample?	Italian palliative care professionals working in hospices, hospital palliative care units and home-based services in Northern Italy. See Table 1 for the breakdown by role, gender, age and experience.	*Methods §2.2; Table 1*
Data collection			
**17**	Interview guide: Were questions, prompts, guides provided by the authors?	Yes. A semi-structured interview guide developed by the research team is provided as Supplementary File S2.	*Supplementary File S2*
**18**	Repeat interviews: Were repeat interviews carried out?	No. Each participant was interviewed once.	*Methods §2.3 — Data collection*
**19**	Audio/visual recording: Did the research use audio or visual recording?	Yes. All interviews were audio-recorded with the participant's written informed consent.	*Methods §2.3 — Data collection*
**20**	Field notes: Were field notes made during and/or after the interview?	Yes. Brief reflexive field notes were taken immediately after each interview to record contextual impressions, salient affective tones and preliminary analytic ideas.	*Methods §2.3 — Data collection*
**21**	Duration: What was the duration of the interviews?	Approximately 60 minutes on average (range ≈45–90 minutes).	*Methods §2.3 — Data collection*
**22**	Data saturation: Was data saturation discussed?	We applied Malterud, Siersma and Guassora's (2016) information power model rather than data saturation in the classical sense. Conceptual richness was considered achieved after sixteen interviews on the basis of the team's iterative analytic meetings.	*Methods §2.2 — Sampling and recruitment*

**23**	Transcripts returned: Were transcripts returned to participants for comment and/or correction?	No. Member checking was deliberately not pursued, consistent with Braun and Clarke's (2019, 2021) epistemological framing of reflexive thematic analysis.	*Methods §2.4 — Data analysis*
**DOMAIN 3: Analysis and findings**			
Data analysis			
**24**	Number of data coders: How many data coders coded the data?	Initial coding was conducted by one member of the research team. Analytic meetings involving the full research team challenged, refined, and reconfigured the developing thematic structure. "Inter-coder reliability" in a positivist sense was not pursued, in keeping with the epistemological framing of reflexive thematic analysis..	*Methods §2.4 — Data analysis*
**25**	Description of the coding tree: Did authors provide a description of the coding tree?	Yes. The thematic structure (four themes and their sub-themes, together with the associated coping function) is reported in the Results section and visualised in Table 2.	*Results; Table 2*
**26**	Derivation of themes: Were themes identified in advance or derived from the data?	Themes were constructed inductively from the data through reflexive thematic analysis. The theoretical literature on coping, emotional labour, compassion fatigue/satisfaction, and meaning-making informed analytic interpretation but did not predetermine the coding frame.	*Methods §2.4 — Data analysis*
**27**	Software: What software, if applicable, was used to manage the data?	No qualitative data analysis software was used. Coding was conducted manually on printed transcripts and supported by structured analytic memos.	*Methods §2.4 — Data analysis*
**28**	Participant checking: Did participants provide feedback on the findings?	No. See item 23 — member checking was not pursued as a deliberate epistemological choice consistent with reflexive thematic analysis.	*Methods §2.4 — Data analysis*
Reporting			
**29**	Quotations presented: Were participant quotations presented to illustrate the themes/findings?	Yes. Verbatim, pseudonymised quotations are presented in the Results section for each theme and sub-theme. To preserve the narrative coherence of individual accounts while protecting confidentiality, each participant was assigned a fixed pseudonym; quotations are attributed by pseudonym together with professional role. Pseudonyms and any contextual details were chosen so as to minimise the risk of re-identification in a small, regionally bounded sample.	*Results*
**30**	Data and findings consistent: Was there consistency between the data presented and the findings?	Yes. Each theme is illustrated by direct participant quotations and discussed in relation to the broader dataset and to the coping framework.	*Results; Discussion*
**31**	Clarity of major themes: Were major themes clearly presented in the findings?	Yes. Four major themes are presented, summarised in Table 2 together with the coping function operating in each sub-theme.	*Results; Table 2*
**32**	Clarity of minor themes: Is there a description of diverse cases or discussion of minor themes?	Yes. Divergent cases (e.g., intrusive identification with the dying vs. integrative growth) were actively sought during analysis; the Discussion considers both trajectories and their co-existence within the same accounts.	*Results §3.4; Discussion §4.1*
