# Supplementary material for: Bearing the Weight of End-of-Life Work: A Qualitative Study of Emotional Experience and Coping Among Palliative Care Professionals in Northern Italy
Source: Healthcare (Basel). 2026 Aug 5;14(15):2415. doi: 10.3390/healthcare14152415 (PMC13464988; doi:10.3390/healthcare14152415)
Supplement: Supplementary file 1 [file healthcare-14-02415-s001.zip › Supplementary File S2_Interview_Guide.pdf]

Supplementary File S2 — Semi-structured Interview Guide

“Bearing the Weight of End-of-Life Work: A Qualitative Study of Emotional Experience and Coping Among Palliative Care Professionals ***in Northern Italy****”*

1. Purpose and Use of the Guide

This semi-structured interview guide was developed by the research team to explore the emotional and experiential dimensions of palliative care work and the coping strategies professionals mobilise to sustain the supporting role. The guide was used flexibly, without a fixed order of questions, in order to favour participants’ expressive freedom and the deepening of significant aspects. Prompts were employed adaptively in response to what each participant raised.

The guide is organised around four thematic areas that mirror the principal constructs in the literature on end-of-life work — moral and decisional experience; psychophysical impact and exposure to suffering and death; relational and emotional dimensions of care; and personal transformations and coping. It opens with a warm-up section to put the participant at ease and closes with a reflective wind-down.

2. Opening of the Interview (≈ 5 minutes)

Before recording: brief restatement of study aims, confidentiality, voluntary nature of participation, right to withdraw at any time, and use of pseudonyms in transcription and publication. Renewed consent for audio-recording. Invitation to ask any questions.

Once recording has started, the interviewer asks an open warm-up question to allow the participant to position themselves in their professional context.

- “Could you tell me briefly about your professional background and the role you currently hold in palliative care?”
- “What initially brought you to palliative care, and what keeps you there today?”

3. Area 1 — Workload, Organisation and Role Boundaries

Aim: to explore the participant’s subjective experience of workload, role demands and structural constraints (theme 1 in the analysis).

Main questions:

- “How would you describe a typical working day in your service?”
- “In your experience, how has the workload changed in recent years? What has changed in particular?”
- “What proportion of your time is spent in direct patient and family care, and what proportion in administrative or organisational tasks?”
- “When you feel overloaded, what do you usually do? What strategies, if any, help you to keep going?”

Probes:

  - “Could you give me a concrete example of a particularly heavy day or week?”
  - “To what extent are these strategies your own, and to what extent are they supported by the team or the institution?”
  - “Are there moments in which you feel that the workload affects the quality of care you can offer?”

4. Area 2 — Relationship with Patients and Families

Aim: to explore the relational and emotional core of palliative care work, including empathic involvement, listening, and the regulation of closeness and distance (theme 2 in the analysis).

Main questions:

- "What does the relationship with patients and their families mean to you?"
- "Could you describe a recent encounter that, in your view, illustrates what is most important in your role?"
- "Are there situations in which empathic involvement feels difficult to manage? How do you handle them?"
- "How do you draw the line between professional involvement and personal involvement?"
- "When you work in the patient's home, what is different from working in a hospice or hospital ward?"

Probes:

  - "What helps you to stay in contact with the patient's suffering without being overwhelmed by it?"
  - "Are there cases that you bring home with you more than others? What do they have in common?"
  - "How do you experience communicating difficult information — for example, breaking bad news or discussing end-of-life choices?"

5. Area 3 — Exposure to Suffering, Loss and Death

Aim: to explore the psychological and existential impact of repeated exposure to suffering and death, including dimensions of moral distress, vicarious trauma, vicarious grief and awareness of mortality (themes 1–4 in the analysis).

Main questions:

- "In your work, how do you experience the proximity to suffering and death?"
- "Have there been situations in which you felt that what you considered the right course of action could not be followed because of institutional, organisational or relational constraints? Could you describe one of these situations?"
- "What is it like, for you, to accompany a patient until the end of their life?"
- "Has working with dying patients changed the way you think about your own life, time and priorities?"
- "Have there been moments in which the loss of a patient produced something similar to grief in you? How do you understand and manage these moments?"

Probes:

  - "Is there a patient — without identifying them — whose story stayed with you in a particularly intense way?"
  - "Do you find yourself identifying with some patients more than with others? What is it about them that triggers identification?"
  - "Has there been any change over time in how you experience death and dying in your work? In what direction?"

6. Area 4 — Coping, Team and Personal Transformations

Aim: to explore the repertoire of coping strategies — individual and team-level — deployed by the participant, including problem-focused, emotion-focused and meaning-focused responses, and to explore personal and professional growth (themes 2–4 in the analysis).

Main questions:

- "When the work becomes emotionally heavy, what helps you to cope? Could you describe two or three concrete strategies?"
- "What role do colleagues and the team play in helping you to manage the emotional load of your work?"
- "In your service, are there structured spaces for supervision, debriefing or psychological support? If yes, how do they work? If no, what would you wish to have?"
- "Outside work, what helps you to recover and to find a sense of balance?"
- "Looking back at your years in palliative care, how have you changed — as a professional and as a person?"
- "In what way, if at all, does your work feel meaningful to you?"

Probes:

  - "Are some of your strategies more focused on solving practical problems, others on managing your emotions, and others on giving meaning to what you do? Could you give an example for each?"
  - "What kind of organisational support do you think your service most needs in order to make your role sustainable in the long term?"
  - "Is there an aspect of your work that, despite the difficulty, you would not change?"

7. Closing of the Interview (≈ 5 minutes)

Reflective wind-down questions:

- "Is there something we have not talked about that you consider important and would like to add?"
- "How was this interview for you? Were there parts that you found particularly meaningful or particularly hard to talk about?"

After recording: thank the participant; remind them of the anonymisation and use of the data; provide a contact for follow-up; if the interview surfaced significant emotional content, share information about institutional/regional services for psychological support; agree on the channel for sharing the published article.

8. Notes for Interviewers

- Maintain an open, non-leading stance; avoid suggesting answers or evaluative reactions.
- Use silence as an active resource; the topic is emotionally charged and participants frequently need time before answering.
- Where a participant becomes visibly upset, pause the interview, check in, and remind them that they can stop at any time.
- Record reflexive field notes immediately after the interview (affective tone, non-verbal aspects, the interviewer's own reactions).
- Where the participant introduces unanticipated themes (e.g., specific moral conflicts, organisational restructurings, COVID-related experiences), follow them; deviating from the guide is encouraged where it serves the research aim.
